# Public health round-up

**DOI:** 10.2471/BLT.19.010719

**Published:** 2019-07-01

**Cholera vaccination campaign**

A health worker dispenses oral cholera vaccine as part of a cholera vaccination campaign in North Kivu in the Democratic Republic of the Congo.

**Figure:**
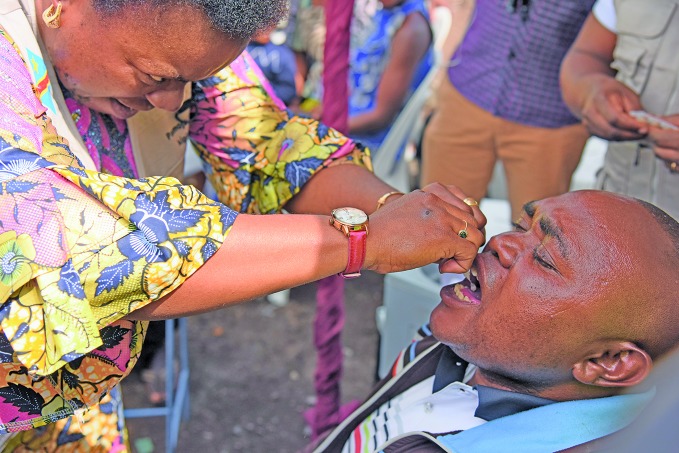
WHO/Eugene Kabambi

## First Ebola case in Uganda

A 5-year-old child from the Democratic Republic of the Congo, who entered Uganda with his parents through the Bwera Border post in the country’s Western Region, was confirmed to have Ebola virus disease on 11 June. 

It was the first confirmed case of Ebola in Uganda, since the Ebola outbreak started in the Democratic Republic of the Congo in August 2018. The case was reported by the Ugandan Ministry of Health and the World Health Organization (WHO). 

The boy was brought into Kagando hospital in the town of Kasese. Health workers at the hospital identified Ebola as a possible cause of illness and the child was transferred to Bwera Ebola Treatment Unit. The confirmation was made at the Uganda Virus Institute.

The Ministry of Health and WHO dispatched a Rapid Response Team to Kasese to identify other people who may be at risk of infection, and to ensure that they are monitored and treated if necessary.

https://afro.who.int/news/confirmation-case-ebola-virus-disease-uganda

## Pakistan HIV outbreak

More than 730 people have been diagnosed with human immunodeficiency virus (HIV) infections in Larkana, Sindh province, southeast Pakistan. Almost 600 of those diagnosed are children. Before the outbreak, some 1200 children were known to be living with HIV throughout Pakistan.

The outbreak was first reported on 25 April 2019. An HIV screening programme was initiated on 28 April, and was expanded on 8 May, with additional health workers being deployed. 

On 16 May, local authorities established a new antiretroviral treatment clinic for children in Larkana. Early last month they were working to ensure supplies of antiretroviral drugs through global procurement processes, as they continued to screen patients and map the disease outbreak.

A team of experts from WHO Headquarters and the Regional Office for the Eastern Mediterranean arrived in Pakistan on 28 May, at the request of the Ministry of Health, to support the response to the outbreak. WHO is coordinating the efforts of an international team, working with local officials.

http://www.emro.who.int/pak/pakistan-news/who-supports-response-to-hiv-outbreak-in-sindh-pakistan.html

## Cholera vaccination

A cholera vaccination campaign launched in North Kivu in the eastern part of the Democratic Republic of the Congo on May 27, reached 762 159 of the targeted 800 000 people within the first week. 

The first of two doses of oral cholera vaccine was administered. At the beginning of June there were tentative plans to conduct the second dose campaign in North Kivu between 9 and 13 July.

The campaign is being implemented by the Ministry of Health with support from WHO and partners and is funded by Gavi, the Vaccine Alliance.

More than 10 000 cases of cholera have been reported in the country since January 2019, leading to more than 240 deaths.

https://afro.who.int/news/major-cholera-vaccination-campaign-begins-north-kivu-democratic-republic-congo

## Ebola transmission easing

Transmission of Ebola virus disease cases in the outbreak in the Democratic Republic of Congo appeared to be easing at the beginning of June, with confirmed cases dropping to around 90 per week at the beginning of the month, down from a peak of 126 cases per week reported in April.

Declines in the incidence of new cases have been most noticeable in hotspots such as Katwa, Mandima and Beni health zones. The outbreak continues to be contained within 12 active health zones in North Kivu and Ituri provinces.

One possible reason for the decline was an improved security situation, which was allowing response teams to implement key interventions including infection prevention and safe burial practices.

As of 6 June 2019, a total of 2025 cases had been reported and 1357 deaths, representing an overall case fatality ratio of 67%. Among those infected were 110 health-care workers, representing 5% of total cases.

https://www.who.int/csr/don/06-june-2019-ebola-drc/en/

## Conflict and mental health

New WHO estimates highlight the need for increased, sustained investment in the development of mental health services in areas affected by conflict. 

According to the estimates published in the *Lancet* on 12 June, roughly one person in five (22%) is living with some form of mental disorder, ranging from mild depression or anxiety to psychosis.

Overall, the mean prevalence was highest for mild mental health conditions (13%), for moderate conditions the prevalence was 4%, and for severe conditions the prevalence was 5%. Depression and anxiety appeared to increase with age in conflict settings and depression was more common among women than men.

The findings suggest that past studies underestimated the burden of mental health conditions in conflict-affected areas, with higher rates of severe mental illness and also of mild to moderate mental health conditions.

http://www.thelancet.com/journals/lancet/article/PIIS0140-6736(19)30934-1/fulltext

## Global epilepsy report

The first global report on epilepsy, entitled, *Epilepsy: a public health imperative *was produced by WHO and key partners and published on 20 June. 

The report highlights available evidence on the burden of epilepsy and the public health response required to address it, and calls for increased action on epilepsy, including the addressing of gaps in research. 

Most people with epilepsy live in low- and middle-income countries and do not have access to treatment. However, effective antiseizure medicines can cost as little as US$ 5 per year and evidence shows that epilepsy interventions can be successfully integrated into primary health care in many countries.

https://www.who.int/mental_health/neurology/epilepsy/report_2019/en

## First self-care guidelines

WHO launched its first consolidated guideline on self-care interventions for sexual and reproductive health on 24 June. 

Entitled *WHO consolidated guidelines on self-care interventions for health: sexual and reproductive health and rights, *the guidelines make evidence-based recommendations on a range of interventions including self-administration of injectable contraception, use of home-based ovulation predictor kits for fertility management, and self-collection of samples for sexually transmitted infection testing.

The guidelines are the first of a planned series, which are expected to cover other health topics as new evidence emerges.

https://www.who.int/reproductivehealth/self-are-interventions/en/

**Cover photo**

A woman returns from her garden near the Mirami point of entry between Uganda and the Democratic Republic of the Congo, where Uganda Red Cross Society supported by the United Nations Children’s Fund and other international agencies, including WHO, are leading an Ebola screening intervention, as part of the response to the ongoing Ebola virus outbreak.

**Figure:**
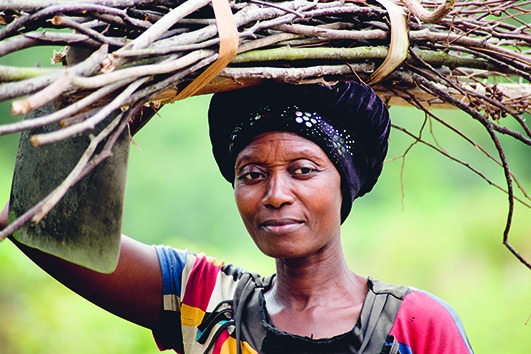
Credit: UNICEF/UN0307465/Bongyereirwe

## Contraception and HIV 

A clinical study supported by WHO and partners and conducted in four African countries found no significant difference in the risk of HIV infection among women using one of three contraceptive methods.

Published in the *Lancet* on 13 June, the Evidence for Contraceptive Options and HIV Outcomes study was done to address concerns raised by evidence from observational studies that use of progestogen-only injectable methods, particularly depo-medroxyprogesterone acetate, might be associated with an increased risk of acquiring HIV.

The study compared three highly effective, reversible methods of contraception (including a non-hormonal method) to evaluate whether there was any difference in the risk of acquiring HIV infection among users of these methods.

http://echo-consortium.com/the-evidence-for-contraceptive-options-and-hiv-outcomes-echo-study-2/

## Food poisoning in Europe

More than 23 million individuals in the WHO European Region become sick from eating contaminated food every year, some 4700 of them dying as a result. Diarrhoeal diseases are responsible for 94% of food-related illnesses, and 63% of food-related deaths.

The data were published in a report entitled *The burden of foodborne diseases in the WHO European Region* and were presented as part of the first-ever World Food Safety Day on 7 June.

An estimated 600 million people, almost 1 in 10 people in the world, fall ill after eating contaminated food and 420 000 die every year.

https://www.who.int/foodsafety/publications/foodborne_disease/fergreport/en/

## Preventing violence against women

WHO, in collaboration with UN Women and 11 partners, launched a new framework for interventions and programmes designed to prevent violence against women. The initiative, called *RESPECT women: preventing violence against women,* was launched on 29 May and is aimed primarily at policy-makers.

About 1 in 3 women worldwide has experienced either physical and/or sexual violence in their lifetime, according to WHO estimates.

https://www.who.int/reproductivehealth/topics/violence/respect-women-framework/en/

**Looking ahead.**

23 September - United Nations High-Level Meeting on universal health coverage. UN Headquarters, New York, United States of America.

24–25 September - Sustainable Development Goals Summit, New York, United States of America.

22–24 October - Fifth High-level Meeting on Transport, Health and Environment, Vienna, Austria.

